# “I had to somehow still be flexible”: exploring adaptations during implementation of brief cognitive behavioral therapy in primary care

**DOI:** 10.1186/s13012-018-0768-z

**Published:** 2018-06-05

**Authors:** Joseph Mignogna, Lindsey Ann Martin, Juliette Harik, Natalie E. Hundt, Michael Kauth, Aanand D. Naik, Kristen Sorocco, Justin Benzer, Jeffrey Cully

**Affiliations:** 1 VISN 17 Center of Excellence for Research on Returning War Veterans, Central Texas VA Health Care System, 4800 Memorial Drive (151C), Waco, TX 76711 USA; 2 Department of Psychiatry and Behavioral Sciences, Texas A&M University Health Science Center, Temple, TX USA; 3 Houston VA Health Services Research and Development Center of Innovations in Quality, Effectiveness, and Safety, Michael E. DeBakey VA Medical Center, Houston, TX USA; 4 Section of Health Services Research, Department of Medicine, Baylor College of Medicine, Houston, TX USA; 5 South Central Mental Illness Research, Education and Clinical Center (SC MIRECC), Houston, TX USA; 6 National Center for Posttraumatic Stress Disorder, Executive Division, White River Junction, VT USA; 7 Department of Psychiatry and Behavioral Sciences, Baylor College of Medicine, Houston, TX USA; 8 Oklahoma City VA Health Care System, Oklahoma City, OK USA; 9 University of Oklahoma Health Sciences Center, Oklahoma City, OK USA; 10 Department of Psychiatry, Dell Medical School, University of Texas, Austin, TX USA

**Keywords:** Cognitive behavioral therapy, Fidelity, Adaptation, Implementation, Integrated primary care, Depression, Anxiety, Qualitative methods, Pragmatic trial

## Abstract

**Background:**

Primary care clinics present challenges to implementing evidence-based psychotherapies (EBPs) for depression and anxiety, and frontline providers infrequently adopt these treatments. The current study explored providers’ perspectives on fidelity to a manualized brief cognitive behavioral therapy (CBT) as delivered in primary care clinics as part of a pragmatic randomized trial. Data from the primary study demonstrated the clinical effectiveness of the treatment and indicated that providers delivered brief CBT with high fidelity, as evaluated by experts using a standardized rating form. Data presented here explore challenges providers faced during implementation and how they adapted nonessential intervention components to make the protocol “fit” into their clinical practice.

**Methods:**

A multiprofessional group of providers (*n* = 18) completed a one-time semi-structured interview documenting their experiences using brief CBT in the primary care setting. Data were analyzed via directed content analysis, followed by inductive sorting of interview excerpts to identify key themes agreed upon by consensus. The Dynamic Adaptation Process model provided an overarching framework to allow better understanding and contextualization of emergent themes.

**Results:**

Providers described a variety of adaptations to the brief CBT to better enable its implementation. Adaptations were driven by provider skills and abilities (i.e., using flexible content and delivery options to promote treatment engagement), patient-emergent issues (i.e., addressing patients’ broader life and clinical concerns), and system-level resources (i.e., maximizing the time available to provide treatment).

**Conclusions:**

The therapeutic relationship, individual patient factors, and system-level factors were critical drivers guiding how providers adapted EBP delivery to improve the “fit” into their clinical practice. Adaptations were generally informed by tensions between the EBP protocol and patient and system needs and were largely not addressed in the EBP protocol itself. Adaptations were generally viewed as acceptable by study fidelity experts and helped to more clearly define delivery procedures to improve future implementation efforts. It is recommended that future EBP implementation efforts examine the concept of fidelity on a continuum rather than dichotomized as adherent/not adherent with focused efforts to understand the context of EBP delivery.

**Trial registration:**

ClinicalTrials.gov, NCT01149772

**Electronic supplementary material:**

The online version of this article (10.1186/s13012-018-0768-z) contains supplementary material, which is available to authorized users.

## Background

Implementing evidence-based psychotherapies (EBPs) is a priority for the largest healthcare organization in the USA, the Veterans Health Administration (VHA). Over the past decade, VHA has heavily invested in implementing and disseminating EBPs for many conditions, including posttraumatic stress disorder, depression, insomnia, and chronic pain [1–7]. However, efforts have largely targeted specialty mental health rather than mental health integrated primary care settings, and challenges remain regarding the broader use of EBPs and whether EBPs are delivered with fidelity [8].

The primary care arena is different in numerous ways from traditional mental health care settings (e.g., faster-paced environment, briefer treatment sessions, briefer courses of treatment) and presents unique challenges to the delivery of EBPs [9]. Brief EBPs have been developed to fit the primary care setting, and preliminary evidence suggests that brief cognitive behavioral therapy (CBT) and problem-solving therapy for depression and anxiety can be effective in primary care clinics [10–12]. Despite the availability of these EBPs that were specifically developed to address known implementation barriers, evidence suggests psychotherapy providers in primary care commonly deliver only isolated components of EBPs [8]. Focused implementation efforts are still needed to improve provider use of EBPs for depression and anxiety.

Providers often struggle to incorporate EBPs that are protocol-based and largely developed in non-clinical settings for research purposes. They often report difficulty “translating” EBP protocols to fit their practice while retaining the empirically supported nature of the treatment itself. Delivering a treatment as it was intended to be delivered versus the need to change the intervention or how it is delivered to improve its “fit” is commonly referred to as fidelity versus adaptation (i.e., flexibility). *Fidelity* refers to how closely a provider skillfully delivers the treatment components believed essential to attaining intended treatment effects [13]. These essential components of treatment are commonly referred to as “core” [14]. Some advocate for a “flexible fidelity,” whereby successful implementation requires providers to deliver an EBP with high fidelity; however, intentional adaptations to “peripheral” components (i.e., any treatment components not viewed as essential to obtaining intended treatment effects) are done to maximize the “fit” of an intervention in the context it is delivered and thereby promote sustainability [15, 16]. The fidelity versus adaptation tension is a notable challenge for all settings but is particularly important for primary care settings where few EBPs currently exist.

This article explores intervention adaptation data from a pragmatic clinical trial of an EBP for integrated primary care settings. Using the Dynamic Adaptation Process (DAP) model, a series of qualitative interviews with mental health providers were conducted to explore their efforts to deliver a brief CBT intervention while balancing the tension between adhering to the treatment protocol and meeting the needs of their patients and clinical settings. Notably, the DAP model is consistent with the “flexible fidelity” view of intervention adaptation [15], highlighting the need to better understand “how to facilitate delivery of EBPs with appropriate adherence and competence, while allowing for adaptations that do not interfere with core elements” ([15], p. 2). The DAP model posits that adaptation occurs across four phases (Exploration, Preparation, Implementation, and Sustainment). During Implementation, five sources of ad hoc adaptations can occur to peripheral components of the treatment or its delivery, namely, patient-emergent issues, provider skills and abilities, available resources, provider knowledge, and organizational changes. Compared to other models examining the fidelity-flexibility tension (e.g., modified fidelity framework [17]), the DAP model provides a meaningful and face-valid model for capturing the tensions our study clinicians reported experiencing in their efforts to balance fidelity with adaptation while implementing brief CBT in primary care.

Brief CBT is designed to better fit the unique needs of the primary care setting. However, implementation challenges remain. Data from this study provide information about the specific ad hoc adaptations clinicians used to implement brief CBT in a mental health integrated primary care setting. Knowledge of the scope of potential adaptations will inform implementation planning for brief EBPs to better balance intervention fidelity with real-world implementation delivery. Additionally, the current study tests the utility of the DAP model for understanding psychotherapy treatment adaptation during implementation_._

## Methods

### Brief cognitive behavioral therapy intervention

The brief CBT intervention was part of a hybrid type 2 study that evaluated the effectiveness and implementation of brief CBT in the primary care setting at two VA medical centers (here referred to as site 1 and site 2 [18]). The intervention targeted primary care patients with heart failure and/or chronic obstructive pulmonary disease, with clinically elevated symptoms of anxiety and/or depression [19–21]. The study used the Reach, Effectiveness, Adoption, Implementation, and Maintenance (RE-AIM) framework to evaluate key effectiveness (e.g., lower rates of depression and anxiety) and implementation outcomes (e.g., fidelity to treatment delivery). Essential, or “core,” components of our brief CBT include (1) delivery of the intervention in four to six sessions, approximately 30–45 min (consistent with the session duration and number of sessions of other EBPs designed for delivery in this setting [10–15]) over a 4-month period and (2) delivery of brief CBT sessions with acceptable adherence and competency scores, as determined through the Adherence and Competence Evaluation (ACE) rating forms described in detail below and attached as an appendix. A minimum of four sessions within 4 months was defined a priori as the criterion for treatment completion. Four sessions allowed providers and patients flexibility to tailor treatment while seeking to deliver care in an efficient manner. Extending therapy beyond six sessions or 4 months was deemed an acceptable treatment adaptation. Importantly, in other research using the parent study data, a dose-response effect from a number of treatment sessions was not found [22].

Sessions 1 and 2 were required modules that explored the connection between physical and mental health problems and improving health through action planning and goal setting. For sessions 3 through 5, providers worked with patients to select three of four skill-focused modules focusing on exercise and nutrition, changing negative thought patterns, and behavioral activation or relaxation. The final treatment session (i.e., session 6) reviewed and consolidated treatment progress. Patients were asked to practice skills between sessions through homework exercises and were offered two monthly “booster calls” following the final session to review and support maintenance of skills acquired during treatment.

Of 180 patients randomized to brief CBT as part of this pragmatic trial, 63.3% (*n* = 114) completed four or more sessions, 51.7% (*n* = 93) completed five or more sessions, and 34.4% (*n* = 62) completed six or more sessions. This represents a higher receipt and number of psychotherapy sessions than are typical for the integrated primary care setting, with prior literature reporting 61% only received one session [8]. Further, given a prior report that only 27% of patients newly diagnosed with depression, anxiety, or posttraumatic stress disorder in the VHA received at least one session of psychotherapy [23], patients’ receipt of psychotherapy in the current study was substantially higher than in typical care. Patients could choose to receive treatment by phone or in-person. Providers typically delivered the first session in person, unless the patient specifically requested a phone session; however, subsequent sessions (i.e., sessions 2–6) were deliverable through either modality. More than half of sessions 2–6 were by phone (60.3%) [21].

Main study outcomes and a complete description of the parent study can be found elsewhere [18, 21]. Additionally, the clinician manual and patient workbook are freely available online [24]. All study procedures were approved by each site’s respective Institutional Review Board and VHA Research & Development Committee.

### Implementation strategy

A multicomponent implementation strategy was guided by the evidence, context, and facilitation domains of the Promoting Action on Research Implementation in Health Services (PARIHS) framework to support adoption of brief CBT. To build providers’ knowledge of brief CBT and its effectiveness (i.e., evidence domain), the implementation strategy included a modular-based online providers’ training program and ongoing audit and feedback from a CBT expert on audio-recorded treatment sessions to promote provider adherence and competence in delivering key module components. Program directors and clinical champions served as internal facilitators supporting and promoting the use of brief CBT in the primary care context. Study team members who were outside the primary care setting provided external facilitation to discuss providers’ challenges implementing brief CBT and problem solve logistical issues through individual and group mentoring meetings with providers [18, 25]. External facilitation allowed the project team to address practice challenges across sites, while internal facilitation provided a detailed within-site approach. Participation in the group mentoring meetings or using session note templates for patients were optional implementation supports available to providers.

Audit and feedback was implemented using ACE rating forms, a standardized evaluation of session audio recordings (see Additional file 1). ACE rating forms provided a checklist to ensure providers’ adequate delivery of core components of each brief CBT session. The CBT expert used these rating forms to inform decisions about overall adherence and skill ratings for each reviewed session [25]. Scores were rated from 1 to 8 for both the Adherence (i.e., delivery of essential components of each treatment session identified on the ACE rating form) and Competence (i.e., ability of the provider to skillfully deliver the core components of the brief CBT manual in a manner that promotes rapport, efficient use of session time, and relevance of the treatment to the patient’s clinical needs) subscales, with 4–5 scores anchored as “moderately” adherent/competent and 6, 7, and 8 classified as “good,” “very good,” and “excellent,” respectively [25].

Consistent with the DAP model [15], providers received ongoing feedback from a CBT expert, using this audit and feedback process, approximately once every 6 months during implementation. In addition to monitoring and providing ongoing feedback to clinicians to promote fidelity to the core treatment components each session, the CBT expert also provided guidance about the acceptability of any treatment adaptations. While scores of 6 or 7 were deemed to be indicative of “high fidelity,” scores of 4 or less were deemed the minimally acceptable ACE rating. On the rare occasion a provider’s ratings fell below a score of 4, additional audit and feedback was provided by the CBT expert until acceptable scale ratings were achieved. The CBT expert permitted and sometimes encouraged providers to adapt peripheral components of the modules to improve the fit of brief CBT into their clinical practice while concurrently maintaining high fidelity to key components. To determine whether a treatment adaptation was acceptable, the CBT expert consulted with the treatment developers. For example, using two sessions to deliver a session module was viewed as acceptable. In contrast, adaptations that interfered with delivery of core treatment components (as detailed for each session in the ACE rating scales; see supplemental material) were viewed as unacceptable. For example, the CBT expert discouraged providers from ancillary discussions and being “off task” (e.g., storytelling by patients) as this detracted from the providers’ time to accomplish essential components of the treatment.

Providers received expert feedback on an average of seven audio recorded sessions. Of 602 audio-recorded treatment sessions, 23% (*n* = 137) were audited [21]. Afterward, providers received feedback by phone and/or email. Feedback was provided in the spirit of professional development and growth rather than an identification of “problematic” or “inappropriate” behaviors. For example, experts attempted to identify positive as well as “developmental” behaviors to encourage growth and development [25]. Providers demonstrated high fidelity ratings, with average adherence and competence of 6.7 and 6.2, respectively [21]. They represented a fairly homogenous group, with limited variability among provider fidelity ratings and no providers scoring less than an average of 6. Further, provider differences (e.g., professional discipline) did not statistically impact treatment outcomes [22].

### Qualitative summative evaluation

#### Data collection

The Consolidated Criteria for Reporting Qualitative Studies guides our reporting of qualitative data collection, analysis, and findings [26]. Providers interviewed were a diverse group of VHA primary care mental health professionals, including licensed psychologists, psychology fellows and interns, licensed clinical social workers, and physician assistants (see Table 1). Providers were directly recruited for the parent study during staff meetings or through contacts in primary care clinics. Providers gave written consent to participate in the parent study and subsequently verbally assented to the summative evaluation qualitative interview. Providers were contacted by email to schedule interviews at a convenient time. One provider did not respond to our request for an interview. Eighteen of the 19 participating providers (95%) completed the interview between November 2012 and April 2014 to document their experiences implementing the brief CBT intervention (*n* = 11 at site 1 and *n* = 7 at site 2). Interviews were conducted over the phone (*n* = 16) or in person (*n* = 2) and were also digitally audio recorded and transcribed. Interviews lasted between 30 and 50 min (*M* = 41 min).

**Table 1:** Provider characteristics

Gender	Female	15 (83.3%)
	Male	3 (16.7%)
Type of provider	Licensed psychologists	5 (27.8%)
	Psychology fellows	6 (33.3%)
	Psychology interns	3 (16.7%)
	Licensed clinical social workers	2 (11.1%)
	Physician assistants	2 (11.1%)
Time as a mental health provider (not including training)	< 1 year	11 (61.1%)
	1–3 years	2 (11.1%)
	4–5 years	3 (16.7%)
	> 10 years	2 (11.1%)
Time affiliated with primary care mental health integration	< 1 year	12 (66.7%)
	1–2 years	6 (33.3%)
Length of time conducting cognitive behavioral therapy in mental health practice (not including training)	< 1 years	13 (72.2%)
	1–3 years	1 (5.6%)
	4–5 years	3 (16.7%)
	> 10 years	1 (5.6%)
Length of time working with chronic and/or complex medically ill patients	< 1 year	9 (50.0%)
	1–3 years	4 (22.2%)
	4–5 years	3 (16.7%)
	6–10 years	1 (5.6%)
	> 10 years	1 (5.6%)

Development of the semi-structured, open-ended interview guides was informed by the PARIHS and RE-AIM frameworks [27–30]. In addition to our use of PARIHS in guiding implementation in the parent study, our rationale for selecting the PARIHS framework to guide the development of the interview guide was influenced by the parsimonious account of factors it offers as important for implementation success. Similarly, RE-AIM offers a parsimonious account of implementation outcomes of interest. This simplicity and strong empirical basis served as the primary reasons we selected the PARIHS framework and RE-AIM model over alternatives [31] to guide the development of our focused yet comprehensive interview guide. Open-ended questions covered the following: (1) providers’ general experiences with the brief CBT intervention, (2) beliefs about evidence-based psychotherapy and manualized treatments, (3) fit of brief CBT in the primary care setting, and (4) perceived outcomes, implementation, and lasting potential of brief CBT (see Additional file 2). A clinical psychologist experienced in qualitative methods (JH), but not directly affiliated with the study, conducted interviews to maintain objectivity in data collection. Providers were interviewed after they completed participation as study therapists. On average, providers interviewed were assigned between 5 and 25 patients (*M* = 9.9 patients) before completing the interview, so their perspectives on implementing brief CBT are based on a broad range of clinical experiences.

#### Analysis

Qualitative analysis occurred in two phases: phase one was largely deductive, and phase two was inductive. In phase one, a medical anthropologist (LM) and a clinical psychologist who served as the study external facilitator (JM) employed directed content analysis [32]. The directed content analysis utilizes predefined, a priori, categories to guide analysis. In this phase of analysis, evidence, context, and facilitation (the main domains of the PARIHS framework), and the Reach, Effectiveness, Adoption, Implementation, and Maintenance domains (from the RE-AIM framework) served as our predefined deductive codes. In addition to these predefined coding categories, analysts also identified additional categories through a posteriori (inductive) coding. Analysts constructed a codebook containing code names and brief descriptions with key elements pertaining to each code. The principal investigator of the parent study (JC) and the interviewer (JH) provided feedback on the codebook, leading to further refinement. Twelve transcripts were coded by both analysts, who regularly met in consensus meetings to compare results, discuss and resolve discrepancies, and revise the codebook. Once the analysts reached consistency in assigning codes and reached consensus on the codebook, they divided the remaining six transcripts and coded independently. Atlas.ti (v. 6.2, Atlas.ti Scientific Software Development GmbH, Berlin, Germany) qualitative software facilitated data management and coding.

In phase two, data deductively coded as “implementation” were extracted from the Atlas.ti database by one analyst (LM) to further explore providers’ perspectives on brief CBT implementation. The analyst (LM) re-read these interview segments and pile sorted them by hand into categories, i.e., subcodes comprising similar topics [33] that emerged inductively from the data. Data coded as “implementation” broadly encompassed aspects of treatment fidelity, for example, level of provider adherence to the treatment protocol, how providers modified and/or deviated from the protocol, and how providers chose to deliver the treatment (i.e., mode of treatment delivery). After further review of the codebook and feedback from the analysis team, it was decided that one of our inductive codes from phase one represented an aspect of implementation, and it, therefore, became a subcode in this phase of our analysis. To optimize validity, the second analyst (JM) audited the findings to offer feedback and suggestions for refinement and to provide consensus on identified subcodes. A detailed review of these subcodes illustrated various ways providers negotiate the tension between fidelity to and a need to display flexibility in delivering brief CBT in the primary care setting and informed our description of the themes below.

## Results

The data collected through the individual qualitative interview data is for the purpose of better understanding providers’ experiences responding to tensions arising from adaptation-fidelity concerns. The following themes detail providers’ experiences navigating the fidelity versus flexibility tension during implementation, including descriptions of adaptations made to peripheral components of the brief CBT or how it was delivered. The DAP model’s provider skills and abilities, patient-emergent issues, and available resources ad hoc adaptations, which occur during the implementation phase, provide an overarching framework to better understand and contextualize these themes [15].

### Adaptations associated with provider skills and abilities

We conceptualized “provider skills and abilities” as a provider’s capacity to adjust his or her approach to delivering brief CBT based on the level or amount of patient engagement with the treatment protocol (i.e., how the provider drew upon his/her clinical skills to *respond* to the patient’s desire to engage in brief CBT). For example, providers describe how some patients required more guidance from providers than others when choosing which of the four skill-building modules (to be used during sessions 3–5) aligned with their treatment goals. One provider took a more hands-on approach by having his/her brief CBT patients read about the modules and then choose what appealed to them. This provider also went on to say that if the patient “didn’t really respond” after reading about the modules, she/he adjusted the approach by talking more with the patient about his/her needs, and then “pushed a little bit more to say, ‘You know…where…do we stand now?’” (K108). Sensing that the patient desired to be more self-directed when selecting modules, another provider described adjusting the approach to a more hands-off style. However, this provider noted that although this patient preferred to “take charge” of module selection, it was atypical for patients to engage with the protocol in this manner (i.e., most providers had to take the more guided hands-on approach) (C12).

One area of brief CBT treatment that posed a challenge to “provider skills and abilities” was patient engagement in the between-session homework exercises. Patients were asked to complete homework exercises to reinforce their skills learned during the sessions, but they did not like this aspect of the brief CBT protocol. Patient dislike of the homework exercises challenged providers’ abilities to deliver effective brief CBT. One provider noted how they set the expectation at the beginning of treatment that homework completion was important for treatment success. However, when patients still did not complete homework, the provider needed to become more “emphatic” with them about the homework assignments as treatment continued (C03). Responding to the non-completion issue, another provider felt a “motivating rationale” was needed to help boost patient engagement with homework (C02).

“Provider skills and abilities” also encompassed a provider’s aptitude to draw on his/her own clinical experiences and adapt module content when needed. For example, providers demonstrated flexibility when delivering module content focused on teaching patients how to do deep breathing exercises. One provider described his/her preference for focused breathing exercises versus the deep breathing approach outlined in the brief CBT protocol. The provider preferred the former approach, noting evidence that deep breathing exercises could trigger a distress response in pulmonary patients. Another provider mentioned adapting *how* breathing was taught by using their “own version,” that is, explaining the skill differently than what was in the providers’ manual (K102). Simplifying language used in the treatment manual, and learning how to better pace content delivered during treatment sessions, also demonstrated providers’ abilities to flexibly adjust to the manualized protocol.

### Adaptations associated with patient-emergent issues

Providers noted how brief CBT sessions did not explicitly offer skill-building modules to address patients’ broader life and clinical concerns such as employment, relationships, finances, traumatic experiences (common among this veteran population), and deaths of friends or family. Because of these issues, patients would often go off topic during brief CBT sessions. Providers reframed or redirected patients back on topic or found ways to acknowledge and address these concerns, in part, if they were brought up in treatment sessions. Providers also incorporated patients’ broader life issues into their brief CBT treatment goals or helped patients connect these other life issues to skills they were learning in the brief CBT modules. Alternatively, providers offered to provide resources for additional help outside the context of the brief CBT intervention (e.g., informing the patient about additional forms of mental health treatment, referring the patient back to the primary care provider to address these concerns), and the option to talk about the issue at the end of the session so as not to dismiss the patient’s real-life concerns. One provider attributed the emergence of these off-topic issues to a simple, non-standardized, quality of life assessment patients completed with their providers during the first session. This assessment asked patients if issues such as physical health, spirituality, finances, relationships, and emotional health are sources of stress, which the provider felt may have signaled to patients that these issues are appropriate topics to talk about during brief CBT.

However, not all issues could be redirected or tied back to patients’ treatment goals. One provider described a pressing issue that required a complete deviation from the brief CBT protocol: “I had a patient whose wife died, and so …I had to step back and do more a … different type of therapy on that particular day” (C09). Providers also reported commonly feeling the need to address patient mental health concerns beyond the intended use of the treatment. For example, one provider felt “constricted” by the manual and described how a patient needed more than what the brief CBT intervention could provide. The patient had posttraumatic stress disorder that caused anxiety and physical symptoms, and the patient’s hypervigilance interfered with his/her ability to go for walks alone. The provider “wanted to spend a little bit more time on that aspect of” the patient’s condition and did so by slightly expanding on the behavioral activation session to help the patient “problem solve around that barrier” (C10).

### Adaptations associated with available resources

Providers made several adaptations to maximize time, which was a valuable resource in the primary care clinics. Brief CBT was designed to be delivered within 35–40 min; in contrast, standard CBT is typically delivered in 45–50 min sessions. Thus, providers pared down session content by focusing on only one teachable skill, summarized portions of module content more thoroughly discussed in the patient treatment manual to ensure that content was understood, and delivered a skills module over two sessions instead of one to adequately cover session content. At the outset of the treatment, the treatment developers determined that, at the clinician’s discretion, a module could be delivered over two sessions instead of one. Consequently, if clinicians did this, it may have reduced the content reviewed during treatment; however, it was viewed as important to the clinician to maintain focus on a particular module over two sessions.

One provider felt it necessary to extend treatment by a few minutes to facilitate the development of the therapeutic relationship for medically and psychologically complicated patients. This provider believed that, although it was possible to deliver session content in the designated timeframe, an “extra ten minutes would just allow me to [say] okay. You know, ‘What are your dogs’ names?’ …. ‘Oh, so your action plan this week is to go out and take Bob and Sue for a walk.’ You know, and so it just brought it [the treatment session] home for them” (K03). Providers also commonly remarked about talkative patients that often wanted to share stories during treatment sessions that shortened the time available to complete session key components. Providers spoke about slight modifications they made during the session to address this scenario in treatment that included asking patients to share these stories at session end, if time remained. Of note, while the brief CBT was designed for delivery in 30 to 45 min, the fidelity rating scales did not explicitly indicate provider strict adherence to this session time limit.

Providers described the difficulty for both providers and patients of being unable to deliver the intervention on a weekly basis (a core component of treatment) when patients no-showed due to life circumstances such as caregiving responsibilities, work, and medical comorbidities. One provider noted: “I think having more time in between sessions makes it harder for them (i.e., patients) to track what’s going on in treatment,” and consequently both providers and patients have “... trouble remembering what we talked about last session” (C06). One provider described backtracking to review material from the previous session, and how “it didn’t feel right because in that first session you’re obviously establishing some level of rapport. …[I]t’s not the same as when I just have somebody (a regular primary care patient) walk into my office. …Because I have to stick to the manual, and there’s certain things I have to say, and certain things I have to get through” (K102).

Block or advanced scheduling was an available resource at site 1 that promoted weekly session delivery, given how busy providers’ schedules were [25], and was part of the implementation strategy. Despite this strategy, if a patient missed an appointment, one provider noted: “[I]t’s hard to get them back in [for the missed treatment session], you know. It would probably take another six weeks before I would have an opening like that available again” (C04). To compensate for missed sessions, providers from site 1 described adding an additional session to the appointment block, booking the patient into a slot normally held for primary care walk-in patients, or scheduling the patient into another slot normally held for something else (e.g., grand rounds).

Delivering brief CBT to patients by phone was a resource providers could utilize during implementation. However, provider viewpoints differed on the impact of phone-based CBT on treatment fidelity. While some providers felt phone-based CBT increased access to care, resulted in fewer distractions during treatment, and was efficient (i.e., cut down on “chit chat” that can occur during in-person visits), others believed adapting brief CBT to phone-based delivery could hinder treatment fidelity. Providers believed seeing the patient in person allowed them to develop a better therapeutic relationship and better assess his/her well-being via nonverbal forms of communication. Phone-based delivery gave providers less flexibility to provide patients with additional copies of worksheets in the treatment workbooks. One patient was asked to move to a more private location due to too many distractions in the background, according to one provider. Some treatment skills were also reported to be more difficult to teach over the phone (e.g., imagery relaxation). One provider articulated how she/he felt patients did not perceive phone sessions as an “official” appointment (some patients were more inclined to miss phone than in-person sessions). Also, providers questioned the efficiency of phone-based brief CBT because the delivery of session content went faster by phone (suggesting less comprehension and integration into a patient’s life) and the belief that more could be accomplished face-to-face where providers could see written homework exercises the patient completed.

## Discussion

This study reports findings from individual qualitative interview data collected from 18 providers describing their experiences with striking a balance between fidelity and flexibility when implementing an EBP (brief CBT) in primary care for medically ill veterans with clinically elevated depression and/or anxiety symptoms. Although providers maintained high fidelity to the brief CBT protocol, they reported challenges and ad hoc adaptations to improve the fit of the treatment for their clinical practice. Interview data supported empirical data that providers were faithful to the brief CBT protocol but reported strong tensions between the need for flexibility while delivering the treatment to ensure effective use in real-world care settings [34]. The delivery of EBPs is complex and involves the need to deliver a treatment according to clinical standards while effectively addressing complex patient needs that are often not directly addressed in the EBP procedures. A critical finding of the current study suggests that the therapeutic relationship, individual patient factors, and system-level factors such as time and scheduling availability are critical drivers that guide provider delivery and adaptations to ensure that EBPs “work” in clinical settings.

Prior literature documents provider modifications to EBP protocols implemented in community or specialty mental health settings [35–37] and other interventions in the primary care setting [38]; however, this is the first study known to the authors to report adaptations made in response to challenges experienced when implementing an EBP in the primary care setting. The DAP model [15] was used to contextualize emergent clinician qualitative themes into ad hoc adaptation categories detailed by the model, namely, provider skills and abilities, patient-emergent issues, and available resources. Prior research investigating adaptations during implementation in primary care of interventions designed to improve health promotion similarly found adaptations based on patient preferences and circumstances [38]. Two DAP model dimensions, provider knowledge and organizational changes, were not identified as sources of adaptation during the implementation of this brief CBT. More research is needed to determine what, if any, impact these sources of adaptation have on implementation of psychotherapy in primary care. Alternative reasons may account for why these areas were not identified. For example, the interview guide did not elicit related provider experiences, or perhaps providers delivering the treatment may not be ideal participants for recognizing and reporting these sources of adaptation.

Providers highlighted challenges they faced in their efforts to implement brief CBT in their practice. They noted the importance of acknowledging or integrating patients’ broader life issues in the context of a focused treatment protocol and accommodating variations in patient engagement with treatment. Also, providers described challenges of learning how to pace brief CBT sessions and treat a complex patient population in the primary care context. Facilitative efforts were designed to ensure core treatment components were delivered while also being flexible to adaptations to accommodate provider styles and clinical needs as they arose. Use of audit and feedback of session audio recordings was well-received by a multi-professional group of providers and appeared to successfully promote the balance between fidelity and flexibility while implementing this treatment.

The therapeutic relationship is likely a key factor guiding when and how providers adapt a treatment protocol to make it “work.” Providers described the challenge of teaching patients tangible skills to promote mental and physical health when faced with a myriad of significant life issues impacting patients’ well-being. Financial struggles, as one of our providers pointed out, is one example of the numerous life issues that may have an impact on patients’ physical and mental health (e.g., Brenk-Franz et al. [39]). The impact of these issues was conceptualized in the development of this brief CBT [18] and cannot be disentangled from treating depression and anxiety. Providers responded to this challenge in diverse ways; however, common to all was the desire to enhance and maintain patient rapport while delivering an EBP during this pragmatic trial.

Study findings have potential implications for future implementation research, as well as policy and planning related to broader dissemination of EBPs to frontline providers. For example, future research about implementing psychotherapy in primary care would benefit from the knowledge that the therapeutic relationship and individual patient factors were critical drivers guiding how providers adapted the treatment protocol to make it “work.” Moreover, dissemination of manualized EBPs requires understanding and addressing the need for providers to modify treatment content and process to meet the unique needs of patients. Chambers and Norton [40] argue that researchers should expect and collect information on treatment adaptations made during implementation and monitor these in terms of impact on individuals, organizations, and communities. The amount of protocol modification is debatable and requires further data and exploration—especially as modification relates to patient outcomes. Although implementation strategies such as fidelity monitoring and provider feedback can be used to reduce treatment “drift,” it is important to acknowledge that fidelity measures typically do not provide an exhaustive list of all prohibited and acceptable behaviors; therefore, fidelity monitoring alone is insufficient for understanding if and how a modification impacts patient outcomes [41, 42]. Applied to the current study, for example, even though providers achieved high fidelity ratings, treatment outcomes may have been even more successful if clinicians had not made any ad hoc adaptations. In fact, a recent meta-analysis found that treatment outcomes were unrelated to provider EBP adherence or competence [43].

Time was found to be a key resource driving adaptations, in terms of best using the scheduled time and in rescheduling patients who miss treatment sessions. Despite early work to design a treatment protocol that works within the demanding primary care environment, contextual issues such as scheduling and the manner in which providers responded to patient no-shows affected their reported ease of adopting the intervention into routine clinical practice. While advanced scheduling of treatment sessions at site 1 promoted weekly delivery of brief CBT, normal interruptions to treatment occurred, for example, patients not showing for an appointment. Given that all patients in this study had a cardiopulmonary condition, it was common for some missed appointments to result from increased physical distress. Providers were challenged to deliver treatment with these missed appointments. The importance of time as a resource may be underappreciated in implementation research. Future efforts to implement brief CBT in primary care may benefit from working with program leadership to anticipate and advise providers as to how to respond to missed appointments (e.g., ensuring providers have open slots in their clinic schedule to accommodate patients’ needs to reschedule appointments in a busy primary care environment).

Although the literature supports the effectiveness of phone-based delivery of EBPs, as well as its ability to reduce barriers and promote engagement in treatment [44, 45], providers in this study voiced some challenges it brings to delivering brief CBT in primary care. For example, some expressed how phone-delivered sessions led to some patients allowing “other things to take precedence” over their treatment. In the current trial, patients were offered treatment in person or by phone. Empirical knowledge is needed to guide provider decisions around how to implement a treatment with this degree of flexibility in treatment delivery to promote high-quality care and address clinically relevant concerns. Given demands for more efficient care delivery, additional research is needed to understand the relationship between treatment modality and treatment fidelity.

Although our study is limited specifically to the primary care mental health integrated setting within the VHA, we believe our findings are transferable to other contexts (i.e., non-VHA settings) where primary care and mental/behavioral health services and providers are integrated. Findings are also transferable more generally to settings where evidence-based practices are implemented by frontline providers who may experience the tension that arises between adaptation and fidelity when delivering these interventions in a real-world clinical context. Our work is limited in that we conducted a summative rather than formative evaluation [46] of providers’ perspectives on fidelity to the brief CBT intervention. Collecting one-time interviews at the end of the study most likely limited the scope of providers’ responses, given the length of time (e.g., many providers delivered care for over 1 year before interview data were collected) they were involved in the project. Important details may have been inadvertently omitted that would have been captured if we had conducted a formative evaluation and captured providers’ viewpoints throughout the phases of our study. Formative-evaluation feedback would have also augmented facilitation efforts by allowing us to use providers’ feedback to make additional modifications to our implementation strategy in real-time. Our study is also limited in that we did not query other key stakeholders in the implementation process, such as clinic directors who could have provided additional insights on the primary care setting and identified possible unknown barriers and facilitators to implementation. While the DAP model served as a useful framework for the current study, one limitation of the model is its inability to clearly articulate overlap between its ad hoc adaptation categories. Qualitative interview data were not always clearly sorted into only one category, and several adaptations likely involve an interaction between the provider’s style and the needs of the patient. Another limitation in the current study is that the reliability of the fidelity assessment method was not evaluated. Future research should look to design study methods that include a reliability assessment of the method used to monitor provider fidelity to the treatment being implemented.

## Conclusions

Providers’ perspectives reveal important insights into challenges of implementing EBPs in a busy primary care setting and highlight tensions between treatment fidelity and ad hoc adaptations made to individualized treatment to address patient needs. Providers’ experiences during our implementation trial highlight potential adaptations to brief CBT that may serve a key role in its successful implementation in the primary care setting. Future efforts to implement EBPs should recognize that adaptations are expected occurrences during treatment delivery and attempt to quantify and categorize adaptations in terms of impact on delivery and, more importantly, on patient outcomes.

## Additional file

Additional file 1: Protocol Adherence and Competency Evaluation (ACE) rating system for rating system for ACCESS (ACE-ACCESS). (PDF 367 kb)

Additional file 2: Clinician Exit Interview Guide. (PDF 166 kb)

## Abbreviations

ACE: Adherence and competence evaluation
CBT: Brief cognitive behavioral therapy
DAP: Dynamic Adaptation Process
EBPs: Evidence-based psychotherapies
PARIHS: Promoting Action on Research Implementation in Health Services
RE-AIM: Reach, Effectiveness, Adoption, Implementation, and Maintenance
VHA: Veterans Health Administration
