# Supplementary material for: “I had to somehow still be flexible”: exploring adaptations during implementation of brief cognitive behavioral therapy in primary care
Source: Implement Sci. 2018 Jun 5;13:76. doi: 10.1186/s13012-018-0768-z (PMC5987469; doi:10.1186/s13012-018-0768-z)
Supplement: Supplementary file 1 — Protocol Adherence and Competency Evaluation (ACE) rating system for rating system for ACCESS (ACE-ACCESS). (PDF 367 kb) [file 13012_2018_768_MOESM1_ESM.pdf]

Protocol Adherence & Competency Evaluation (ACE) Rating System for ACCESS (ACE-ACCESS)

Clinician #:	Patient #:
ACE Rater:	
Date:	

Modules taught or reviewed (check all that apply):

______ Core Session 1

______ Core Session 2

______ Module A: Physical Health

______ Module B: Changing Thoughts

______ Module C: Behavioral Activation

______ Module D: Learning to Relax

______ Session 6 and Wrap-Up

KEEP BLANK

ACE Rating Form 1 (Core Session 1): AUDIT FORM

**CORE SESSION 1: ADHERENCE**<br>**Adherence involves following the treatment protocol. Overall adherence is not based on the number of "YES" answers circled below only, but overall performance. Adherence is TO the manual.**			**CORE SESSION 1: SKILL**<br>**The following are constructs related to being skilled. Skill does not require all options be met, but is based on 0-8 rating as defined below. Skill includes rapport building and procedural techniques. Skill is use OF the manual.**
Introduced self and the ACCESS Program	YES	NO	RAPPORT TECHNIQUES:
Discussed the purpose and rationale for the intervention including how it is intended to help and discussion of confidentiality and limits	YES	NO	(1) Attended to the needs of the patient while covering topics of the treatment manual<br>(2) Used language that the patient could follow and understand
Introduced the patient to the ACCESS materials and procedures	YES	NO	(3) Built a trusting relationship with the patient
Learned from the patient about their chronic condition and feelings of stress and negative emotions	YES	NO	(4) Answered patient questions and concerns<br>PROCEDURAL TECHNIQUES:
Discussed the overlap between physical and emotional health including the idea of self-management	YES	NO	(1) Set the agenda with the patient at the start of the session
Discussed quality of life including Quality of Life Worksheets, most pressing concerns, and strategies for improving QOL	YES	NO	(2) Was successful in setting goals and action plans with the patient
Reviewed the home practice assignment for the coming week	YES	NO	(3) Made good transitions while using the manual
Wrapped up the session and discussed next meeting and telephone calls	YES	NO	(4) Identified examples and assignments that matched patient needs<br>(5) Utilized worksheets in session to facilitate session potency

Overall ADHERENCE Rating: (Please circle the most fitting score 0-8)

0	1	2	3	4	5	6	7	8
**VERY POOR**<br>**(no reference/ non-use of manual)**<br>**0% time on task**		**POOR**<br>**(minimal/infrequent use of manual)**<br>**25% time on task**		**Moderate**<br>**(adequate use of manual)**<br>**50% time on task**		**GOOD**<br>**(frequent use of manual)**<br>**75% time on task**		**VERY GOOD/ EXCELLENT**<br>**(near complete use of manual and materials)**<br>**≥95% time on task**

**ADHERENCE VALIDITY RATING – Was the Adherence rating valid?**	**YES**	**NO**	**If NO, please explain on the "Feedback Form"**

Overall SKILL Rating:

0	1	2	3	4	5	6	7	8
**VERY POOR**<br>**(no rapport and/or procedural technique)**		**POOR**<br>**(limited rapport and/or procedural technique)**		**Moderate**<br>**(adequate rapport and/or procedural technique)**		**GOOD**<br>**(consistent rapport and/or procedural technique)**		**VERY GOOD/ EXCELLENT**<br>**(outstanding rapport and/or procedural technique throughout)**

ACE Rating Form 1 (Core Session 1): FEEDBACK FORM

NOTABLE SESSION OCCURANCES (e.g. patient emergencies or other significant issues that may have impacted the session should be listed here; also please list any other important session information here):

____

____

____

____

SESSION STRENGTHS:____

____

____

____

____

AREAS FOR IMPROVEMENT:____

____

____

____

____

ACE Rating Form 2 (Core Session 2): AUDIT FORM

CORE SESSION 2: ADHERENCE<br>Adherence involves following the treatment protocol. Overall adherence is not based on the number of “YES” answers circled below only, but overall performance. Adherence is TO the manual.		
Reviewed home practice assignments including: personal meaning of having a chronic illness; past and current self-management approach	YES	NO
Set initial goals with patient including developing broad or general goals as a foundation for more detailed action plans in future sessions	YES	NO
Discussed intervention options and matched these to the individual patient needs	YES	NO
Discussed Action Plans	YES	NO
Reviewed the home practice assignment for the coming week: applying and monitoring a first action plan	YES	NO
Wrapped up the session and discussed next meeting and telephone calls	YES	NO

CORE SESSION 2: SKILL
The following are constructs related to being skilled. Skill does not require all options be met, but is based on 0-8 rating as defined below. Skill includes rapport building and procedural techniques. Skill is use OF the manual.

RAPPORT TECHNIQUES:

(1) Attended to the needs of the patient while covering topics of the treatment manual

(2) Used language that the patient could follow and understand

(3) Built a trusting relationship with the patient

(4) Answered patient questions and concerns

PROCEDURAL TECHNIQUES:

(1) Set the agenda with the patient at the start of the session

(2) Was successful in setting goals and action plans with the patient

(3) Made good transitions while using the manual

(4) Identified examples and assignments that matched patient needs

(5) Utilized worksheets in session to facilitate session potency

Overall ADHERENCE Rating: (Please circle the most fitting score 0-8)

0	1	2	3	4	5	6	7	8
**VERY POOR**<br>(no reference/ non-use of manual)<br>0% time on task		**POOR**<br>(minimal/infrequent use of manual)<br>25% time on task		**Moderate**<br>(adequate use of manual)<br>50% time on task		**GOOD**<br>(frequent use of manual)<br>75% time on task		**VERY GOOD/ EXCELLENT**<br>(near complete use of manual and materials)<br>$\geq$95% time on task

ADHERENCE VALIDITY RATING – Was the Adherence rating valid? YES NO **If NO, please explain on the “Feedback Form”**

Overall SKILL Rating:

0	1	2	3	4	5	6	7	8
**VERY POOR**<br>(no rapport and/or procedural technique)		**POOR**<br>(limited rapport and/or procedural technique)		**Moderate**<br>(adequate rapport and/or procedural technique)		**GOOD**<br>(consistent rapport and/or procedural technique)		**VERY GOOD/ EXCELLENT**<br>(outstanding rapport and/or procedural technique throughout)

ACE Rating Form 2 (Core Session 2): FEEDBACK FORM

NOTABLE SESSION OCCURANCES (e.g. patient emergencies or other significant issues that may have impacted the session should be listed here; also please list any other important session information here):

____

____

____

____

SESSION STRENGTHS: ____

____

____

____

____

AREAS FOR IMPROVEMENT: ____

____

____

____

____

ACE Rating Form 3 (Module A: Physical Health): AUDIT FORM

MODULE A: ADHERENCE<br>Adherence involves following the treatment protocol. Overall adherence is not based on the number of "YES" answers circled below only, but overall performance. Adherence is TO the manual.		
Reviewed home practice including discussion of patient's reactions to assignment, use of skills, and any barriers	YES	NO
Reviewed Module A goals and skills including living healthy and managing illness to improve physical health and quality of life	YES	NO
Discussed concept of self-management and goal setting	YES	NO
Reviewed what patient has done in the past and currently to improve physical health	YES	NO
Facilitated the patient's selection of individual skills to work on	YES	NO
Administered/reviewed selected Module A skills * (see additional page for details on specific sub-module coverage) *	YES	NO
Set concrete goals including identifying areas for change and formulated an action plan with the patient	YES	NO
Reviewed home practice assignment for the coming week: implementing an action plan and monitoring management of physical health	YES	NO
Wrapped up the session and discussed next meeting and telephone calls	YES	NO

MODULE A: SKILL
The following are constructs related to being skilled. Skill does not require all options be met, but is based on 0-8 rating as defined below. Skill includes rapport building and procedural techniques. Skill is use OF the manual.

RAPPORT TECHNIQUES:

(1) Attended to the needs of the patient while covering topics of the treatment manual

(2) Used language that the patient could follow and understand

(3) Built a trusting relationship with the patient

(4) Answered patient questions and concerns

PROCEDURAL TECHNIQUES:

(1) Set the agenda with the patient at the start of the session

(2) Was successful in setting goals and action plans with the patient

(3) Made good transitions while using the manual

(4) Identified examples and assignments that matched patient needs

(5) Utilized worksheets in session to facilitate session potency

Overall ADHERENCE Rating: (Please circle the most fitting score 0-8)

0	1	2	3	4	5	6	7	8
**VERY POOR**<br>(no reference/ non-use of manual)<br>0% time on task		**POOR**<br>(minimal/infrequent use of manual)<br>25% time on task		**Moderate**<br>(adequate use of manual)<br>50% time on task		**GOOD**<br>(frequent use of manual)<br>75% time on task		**VERY GOOD/ EXCELLENT**<br>(near complete use of manual and materials)<br>≥95% time on task

ADHERENCE VALIDITY RATING – Was the Adherence rating valid? | YES | NO | **If NO, please explain on the "Feedback Form"**

Overall SKILL Rating:

0	1	2	3	4	5	6	7	8
**VERY POOR**<br>(no rapport and/or procedural technique)		**POOR**<br>(limited rapport and/or procedural technique)		**Moderate**<br>(adequate rapport and/or procedural technique)		**GOOD**<br>(consistent rapport and/or procedural technique)		**VERY GOOD/ EXCELLENT**<br>(outstanding rapport and/or procedural technique throughout)

ACE Rating Form 3 (Module A: Physical Health): FEEDBACK FORM

NOTABLE SESSION OCCURANCES (e.g. patient emergencies or other significant issues that may have impacted the session should be listed here; also please list any other important session information here):

____

____

____

____

SESSION STRENGTHS:______________________________

____

____

____

____

AREAS FOR IMPROVEMENT:____________________________

____

____

____

____

ACE Rating Form 4 (Module B: Changing Thoughts): AUDIT FORM

MODULE B: ADHERENCE<br>Adherence involves following the treatment protocol. Overall adherence is not based on the number of "YES" answers circled below only, but overall performance. Adherence is TO the manual.			MODULE B: SKILL<br>The following are constructs related to being skilled. Skill does not require all options be met, but is based on 0-8 rating as defined below. Skill includes rapport building and procedural techniques. Skill is use OF the manual.
Reviewed home practice including discussion of patient's reactions to assignment, use of skills, and any barriers	YES	NO	RAPPORT TECHNIQUES:
Discussed why the patient chose this module	YES	NO	(1) Attended to the needs of the patient while covering topics of the treatment manual
Discussed ways thoughts relate to mood and functioning	YES	NO	(2) Used language that the patient could follow and understand
Talked with patient about identifying negative thoughts	YES	NO	(3) Built a trusting relationship with the patient
Began working with patient to change thought patterns including modifying negative thoughts and utilizing coping self-statements	YES	NO	(4) Answered patient questions and concerns<br>PROCEDURAL TECHNIQUES:
Set concrete goals including identifying areas for change and formulating an action plan	YES	NO	(1) Set the agenda with the patient at the start of the session<br>(2) Was successful in setting goals and action plans with the patient
Reviewed the home practice assignment for the coming week : monitoring thoughts and increasing the use of coping self-statements	YES	NO	(3) Made good transitions while using the manual<br>(4) Identified examples and assignments that matched patient needs
Wrapped up the session and discussed next meeting and telephone calls	YES	NO	(5) Utilized worksheets in session to facilitate session potency

Overall ADHERENCE Rating: (Please circle the most fitting score 0-8)

0	1	2	3	4	5	6	7	8
VERY POOR<br>(no reference/ non-use of manual)<br>0% time on task		POOR<br>(minimal/infrequent use of manual)<br>25% time on task		Moderate<br>(adequate use of manual)<br>50% time on task		GOOD<br>(frequent use of manual)<br>75% time on task		VERY GOOD/ EXCELLENT<br>(near complete use of manual and materials)<br>≥95% time on task

ADHERENCE VALIDITY RATING – Was the Adherence rating valid? YES NO **If NO, please explain on the "Feedback Form"**

Overall SKILL Rating:

0	1	2	3	4	5	6	7	8
VERY POOR<br>(no rapport and/or procedural technique)		POOR<br>(limited rapport and/or procedural technique)		Moderate<br>(adequate rapport and/or procedural technique)		GOOD<br>(consistent rapport and/or procedural technique)		VERY GOOD/ EXCELLENT<br>(outstanding rapport and/or procedural technique throughout)

ACE Rating Form 4 (Module B: Changing Thoughts): FEEDBACK FORM

NOTABLE SESSION OCCURANCES (e.g. patient emergencies or other significant issues that may have impacted the session should be listed here; also please list any other important session information here):

____

____

____

____

SESSION STRENGTHS:____

____

____

____

____

AREAS FOR IMPROVEMENT:____

____

____

____

____

ACE Rating Form 5 (Module C: Behavioral Activation): AUDIT FORM

MODULE C: ADHERENCE<br>Adherence involves following the treatment protocol. Overall adherence is not based on the number of "YES" answers circled below only, but overall performance. Adherence is TO the manual.		
Reviewed home practice including discussion of patient's reactions to assignment, use of skills, and any barriers	YES	NO
Discussed why the patient chose this module	YES	NO
Introduced the concept of increasing pleasant activities and behavioral activation	YES	NO
Introduced concept of monitoring behavior and mood	YES	NO
Facilitated patient identification of activities to improve quality of life	YES	NO
Set concrete goals including identifying areas for change and formulating an action plan	YES	NO
Reviewed the home practice assignment for the coming week : applying and monitoring behavioral activation skills	YES	NO
Wrapped up the session and discussed next meeting and telephone calls	YES	NO

MODULE C: SKILL<br>The following are constructs related to being skilled. Skill does not require all options be met, but is based on 0-8 rating as defined below. Skill includes rapport building and procedural techniques. Skill is use OF the manual.
RAPPORT TECHNIQUES:
(1) Attended to the needs of the patient while covering topics of the treatment manual
(2) Used language that the patient could follow and understand
(3) Built a trusting relationship with the patient
(4) Answered patient questions and concerns
PROCEDURAL TECHNIQUES:
(1) Set the agenda with the patient at the start of the session
(2) Was successful in setting goals and action plans with the patient
(3) Made good transitions while using the manual
(4) Identified examples and assignments that matched patient needs
(5) Utilized worksheets in session to facilitate session potency

Overall ADHERENCE Rating: (Please circle the most fitting score 0-8)

0	1	2	3	4	5	6	7	8
**VERY POOR**<br>(no reference/ non-use of manual)<br>0% time on task		**POOR**<br>(minimal/infrequent use of manual)<br>25% time on task		**Moderate**<br>(adequate use of manual)<br>50% time on task		**GOOD**<br>(frequent use of manual)<br>75% time on task		**VERY GOOD/ EXCELLENT**<br>(near complete use of manual and materials)<br>$\geq$95% time on task

ADHERENCE VALIDITY RATING – Was the Adherence rating valid? YES | NO **If NO, please explain on the "Feedback Form"**

Overall SKILL Rating:

0	1	2	3	4	5	6	7	8
**VERY POOR**<br>(no rapport and/or procedural technique)		**POOR**<br>(limited rapport and/or procedural technique)		**Moderate**<br>(adequate rapport and/or procedural technique)		**GOOD**<br>(consistent rapport and/or procedural technique)		**VERY GOOD/ EXCELLENT**<br>(outstanding rapport and/or procedural technique throughout)

ACE Rating Form 5 (Module C: Behavioral Activation): FEEDBACK FORM

NOTABLE SESSION OCCURANCES (e.g. patient emergencies or other significant issues that may have impacted the session should be listed here; also please list any other important session information here):

SESSION STRENGTHS:

AREAS FOR IMPROVEMENT:

ACE Rating Form 6 (Module D: Learning to Relax): AUDIT FORM

MODULE D: ADHERENCE<br>Adherence involves following the treatment protocol. Overall adherence is not based on the number of "YES" answers circled below only, but overall performance. Adherence is TO the manual.		
Reviewed home practice including discussion of patient's reactions to assignment, use of skills, and any barriers	YES	NO
Discussed why the patient chose this module	YES	NO
Introduced the concept of worry, stress and anxiety	YES	NO
Discussed the purpose of relaxation exercises	YES	NO
Facilitated patient learning how to relax using diaphragmatic deep breathing and relaxed posture	YES	NO
Facilitated patient learning how to relax using imagery	YES	NO
Facilitated patient using techniques to help control emotional and physical sensations of tension	YES	NO
Set concrete goals including identifying areas for change and formulating an action plan	YES	NO
Reviewed the home practice assignment for the coming week : applying and monitoring relaxation skills	YES	NO
Wrapped up the session and discussed next meeting and telephone calls	YES	NO

MODULE D: SKILL
The following are constructs related to being skilled. Skill does not require all options be met, but is based on 0-8 rating as defined below. Skill includes rapport building and procedural techniques. Skill is use OF the manual.

RAPPORT TECHNIQUES:

(1) Attended to the needs of the patient while covering topics of the treatment manual

(2) Used language that the patient could follow and understand

(3) Built a trusting relationship with the patient

(4) Answered patient questions and concerns

PROCEDURAL TECHNIQUES:

(1) Set the agenda with the patient at the start of the session

(2) Was successful in setting goals and action plans with the patient

(3) Made good transitions while using the manual

(4) Identified examples and assignments that matched patient needs

(5) Utilized worksheets in session to facilitate session potency

Overall ADHERENCE Rating: (Please circle the most fitting score 0-8)

0	1	2	3	4	5	6	7	8
**VERY POOR**<br>(no reference/ non-use of manual)<br>0% time on task		**POOR**<br>(minimal/infrequent use of manual)<br>25% time on task		**Moderate**<br>(adequate use of manual)<br>50% time on task		**GOOD**<br>(frequent use of manual)<br>75% time on task		**VERY GOOD/ EXCELLENT**<br>(near complete use of manual and materials)<br>≥95% time on task

ADHERENCE VALIDITY RATING – Was the Adherence rating valid? YES NO **If NO, please explain on the "Feedback Form"**

Overall SKILL Rating:

0	1	2	3	4	5	6	7	8
**VERY POOR**<br>(no rapport and/or procedural technique)		**POOR**<br>(limited rapport and/or procedural technique)		**Moderate**<br>(adequate rapport and/or procedural technique)		**GOOD**<br>(consistent rapport and/or procedural technique)		**VERY GOOD/ EXCELLENT**<br>(outstanding rapport and/or procedural technique throughout)

ACE Rating Form 6 (Module D: Learning to Relax): FEEDBACK FORM

NOTABLE SESSION OCCURANCES (e.g. patient emergencies or other significant issues that may have impacted the session should be listed here; also please list any other important session information here):

____

____

____

____

SESSION STRENGTHS: ____

____

____

____

____

AREAS FOR IMPROVEMENT: ____

____

____

____

____

ACE Rating Form 7 (Core Session 6: Wrap-up): AUDIT FORM

**CORE SESSION 6: ADHERENCE**<br>**Adherence involves following the treatment protocol. Overall adherence is not based on the number of "YES" answers circled below only, but overall performance. Adherence is TO the manual.**		
Reviewed home practice including discussion of patient's reactions to assignment, use of skills, and any barriers	YES	NO
Reviewed patient progress in treatment including patient skill preferences ,use of skills, and impact on quality of life and functioning	YES	NO
Discussed how to maintain changes including addressing barriers and accessing motivators for change	YES	NO
Discussed with patient when to seek out additional help	YES	NO
Wrap-up and closing of the treatment	YES	NO
Scheduled the first follow-up call for two weeks	YES	NO

CORE SESSION 6: SKILL
The following are constructs related to being skilled. Skill does not require all options be met, but is based on 0-8 rating as defined below. Skill includes rapport building and procedural techniques. Skill is use OF the manual.

RAPPORT TECHNIQUES:

(1) Attended to the needs of the patient while covering topics of the treatment manual

(2) Used language that the patient could follow and understand

(3) Built a trusting relationship with the patient

(4) Answered patient questions and concerns

PROCEDURAL TECHNIQUES:

(1) Set the agenda with the patient at the start of the session

(2) Was successful in setting goals and action plans with the patient

(3) Made good transitions while using the manual

(4) Identified examples and assignments that matched patient needs

(5) Utilized worksheets in session to facilitate session potency

Overall ADHERENCE Rating: (Please circle the most fitting score 0-8)

0	1	2	3	4	5	6	7	8
**VERY POOR**<br>(no reference/ non-use of manual)<br>0% time on task		**POOR**<br>(minimal/infrequent use of manual)<br>25% time on task		**Moderate**<br>(adequate use of manual)<br>50% time on task		**GOOD**<br>(frequent use of manual)<br>75% time on task		**VERY GOOD/ EXCELLENT**<br>(near complete use of manual and materials)<br>≥95% time on task

ADHERENCE VALIDITY RATING – Was the Adherence rating valid? YES NO **If NO, please explain on the "Feedback Form"**

Overall SKILL Rating:

0	1	2	3	4	5	6	7	8
**VERY POOR**<br>(no rapport and/or procedural technique)		**POOR**<br>(limited rapport and/or procedural technique)		**Moderate**<br>(adequate rapport and/or procedural technique)		**GOOD**<br>(consistent rapport and/or procedural technique)		**VERY GOOD/ EXCELLENT**<br>(outstanding rapport and/or procedural technique throughout)

ACE Rating Form 7 (Core Session 6: Wrap-up): FEEDBACK FORM

NOTABLE SESSION OCCURANCES (e.g. patient emergencies or other significant issues that may have impacted the session should be listed here; also please list any other important session information here):

____

____

____

____

SESSION STRENGTHS: ____

____

____

____

____

AREAS FOR IMPROVEMENT: ____

____

____

____

____
