# Supplementary material for: “I had to somehow still be flexible”: exploring adaptations during implementation of brief cognitive behavioral therapy in primary care
Source: Implement Sci. 2018 Jun 5;13:76. doi: 10.1186/s13012-018-0768-z (PMC5987469; doi:10.1186/s13012-018-0768-z)
Supplement: Supplementary file 2 — Clinician Exit Interview Guide. (PDF 166 kb) [file 13012_2018_768_MOESM2_ESM.pdf]

Clinician Exit Interview Guide

Participation with ACCESS* (data provided by interviewer)

Our records show that you:

Participated in the ACCESS study from _________ until ___________.

Were assigned a total of ____________ ACCESS patients in total.

General Questions about your experiences with the ACCESS trial

1) Why did you decide to participate in the ACCESS trial?

2) Overall, how would you describe your experience as an ACCESS clinician?

3) Did using ACCESS impact or change the care you provided in your PC-MHI clinic? If so, how? Did using ACCESS impact your professional development? If so, how?

4) Did you encounter any concerns, challenges, or barriers to your professional development while using ACCESS in PC-MHI? Please explain. Did you encounter any concerns, challenges, or barriers to the care you provided patients or to the use of ACCESS in your PC-MHI clinic? Please explain.

Importance of ACCESS, Evidence Supporting ACCESS

5) In your opinion, how important is using brief psychotherapies, like brief CBT (ACCESS), in providing treatment for your PC-MHI patients?

6) Prior to enrolling in the ACCESS trial, what were your thoughts about using manualized therapies in PC-MHI?

7) Did your participation in the ACCESS trial change your thoughts about using manualized therapies in PC-MHI? If so, how?

8) Do you consider the ACCESS program to be evidence-based? Please explain.

Fit of ACCESS in PC-MHI

9) How does the ACCESS program fit within the overall mission of the PC-MHI clinic?

10) Now I'd like to ask about some specific components and aspects of the ACCESS intervention and get your perspectives about how well these fit with your PC-MHI practice:

- General content of the intervention:
- Patient-centeredness (ability and flexibility to meet the unique needs of your patients):
- Session length:
- Session frequency:

- Overall treatment duration:
- Modular nature of the treatment:
- Telephone sessions:

11) In your opinion, is there sufficient organizational infrastructure at your PC-MHI clinic (e.g. staff, clinic space, leadership, administration, scheduling procedures, procedure for coordinating care, etc.) to support treatment initiatives such as ACCESS?

12) Outside of the ACCESS program, did or do you regularly provide psychotherapy in PC-MHI?

If yes, continue to Q13
If no, Skip ahead to Q16

13) What does this psychotherapy typically looks like in terms of types of typical treatments used, session length/frequency, and duration of treatment?

14) What are some of the challenges to providing psychotherapy in PC-MHI?

15) Did the ACCESS program address any of the challenges you just listed? If so, how?

16) In your opinion is psychotherapy in PC-MHI currently being utilized as frequently or as effectively as it could be? Please explain.

<u>ACCESS Outcomes</u>

17) Do you feel your site's involvement ACCESS program increased or improved the availability of psychotherapy in your PC-MHI clinic? If so, how? (reach)

18) In your opinion, was ACCESS an effective treatment for the patients you treated with this program?

19) What impact do you believe ACCESS had on your patient's emotional health? (effectiveness)

20) What impact do you believe ACCESS had on your patient's physical health? (effectiveness)

21) Were there certain patients that tended to respond better or worse to the treatment? (effectiveness)

22) In general, how well do you feel the ACCESS program was received by PC-MHI providers at your site? (adoption)

23) Did you feel adequately prepared and supported to use the ACCESS program? (fidelity)

24) How easy or challenging was it to adhere to the intervention procedures? (fidelity)

25) Did you find that you tended to alter the administration of ACCESS in any way? If so, how?

26) ACCESS used several strategies to support clinicians – e.g. online training, regular facilitation meetings, and CBT expert feedback – what are your thoughts about these services? Are there other services that you think would have been helpful to provide to clinicians?

27) Did you consult/share with colleagues or supervise trainees in the use of ACCESS?

28) Aside from the impact of ACCESS on your practice and your patients, can you identify any other "ripple effects" of the ACCESS program? For example, clinic system, clinic culture, impact on trainees, etc.?

29) How and to what extent do you intend to use the ACCESS program or any of its components in the future? (maintenance)

30) Do you believe there will be any "carry over" effects on your PC-MHI clinic once the ACCESS trial has ended? (maintenance)

* The brief Cognitive Behavioral Therapy Intervention was referred to using the acronym: Adjusting to Chronic Conditions with Education, Support, and Skills
